# High-fat diet causes undesirable bone regeneration by altering the bone marrow environment in rats

**DOI:** 10.3389/fendo.2023.1088508

**Published:** 2023-03-28

**Authors:** Feiyu Cai, Aihemaitijiang Yusufu, Kai Liu, Wenjiao Chen, Ruomei Zhao, Yanshi Liu, Yi Liu

**Affiliations:** ^1^ Department of Burns and Plastic Surgery & Wound Repair Surgery, The Lanzhou University Second Hospital, Lanzhou, Gansu, China; ^2^ Department of Trauma and Micro Reconstructive Surgery, The First Affiliated Hospital of Xinjiang Medical University, Urumqi, China; ^3^ Department of Orthopaedics, The Affiliated Hospital of Southwest Medical University, Luzhou, Sichuan, China

**Keywords:** adipose tissue, bone regeneration, distraction osteogenesis, high-fat-diet, obesity

## Abstract

**Objective:**

Diet structure has changed greatly over the last few decades, and high-calorie diets have become an integral part of people’s daily diet, as well as the important cause of obesity in society. Several organ systems, including the skeletal system, are seriously affected by high-fat-diets (HFD) in the world. There is, however, still a lack of knowledge about the effects of HFD on bone regeneration and the possible mechanisms involved. In this study, the difference in bone regeneration between rats under HFD and low-fat-diets (LFD) was evaluated by monitoring the process of bone regeneration in distraction osteogenesis (DO) model animals, as well as the possible mechanisms.

**Methods:**

A total of 40 Sprague Dawley (SD) rats (5 weeks old) were randomly divided into HFD group (n=20) and LFD group (n=20). Except for feeding methods, there were no differences between the two groups in terms of treatment conditions. All animals received the DO surgery eight weeks after starting to feed. After a delay of 5 days (latency phase), the active lengthening phase was performed for 10 days (0.25 mm/12 h), and the consolidation phase followed for 42 days. An observational study of bone included radioscopy (once a week), micro-computed tomography (CT), general morphology, biomechanics, histomorphometry, and immunohistochemistry.

**Result:**

The results showed that HFD group had a higher body weight than LFD group after 8, 14, and 16 weeks of feeding. Furthermore, at the final observation, there were statistically significant differences between LFD group and HFD group in terms of total cholesterol (TC), triglycerides (TG), low-density lipoprotein (LDL), and high-density lipoprotein (HDL) levels. Additionally, observations on bone regeneration showed a slower regeneration and a lower biomechanical strength in HFD group than LFD group, based on radiography, micro-CT, general morphology, biomechanics, histomorphometry, and immunohistochemistry.

**Conclusion:**

In this study, HFD resulted in elevated blood lipids, increased adipose differentiation at the bone marrow level, and delayed bone regeneration. The pieces of evidence are beneficial to better understand the association between diet and bone regeneration and to adjust the diet optimally for fracture patients.

## 1 Introduction

Over the years, diet-induced obesity has become more common in society, and researchers have paid a lot of attention to it. It is well known that a habit of high-fat diet (HFD) has a profound impact on the metabolism of various organ systems in the human body, including the skeletal system. HFD and bone, however, have been the subject of widespread controversy, with many researchers coming to different conclusions about the effects of HFD on bones. According to a widely accepted view in past research, high body mass was associated with increased mechanical loading, which could benefit bone health (1–3). However, recent evidence suggests that children and adolescents who were obese were more likely to suffer from bone fractures (4). According to Hou J et al., there is also a complex link between obesity and bone health through different mechanisms that may be involved in leptin, adiponectin, and many pro-inflammatory factors (5).

Some related studies have observed that mice fed with a HFD suffer from low bone mass, but the changes in osteoclast and osteoblast function were not always consistent *in vitro* (6–8). Generally, low bone mass is associated with an increase in osteoclasts and a decrease in osteoblasts. However, there are a number of hormones and circulating cytokines involved in regulating the formation and apoptosis of osteoblasts and osteoclasts, which is an extremely complex process. (5, 9–11). Moreover, bone marrow mesenchymal stem cells (BMSCs) can differentiate into osteoblasts or adipocytes (12, 13). Hence, osteogenesis may be decreased by excessive differentiation of BMSCs into bone marrow adipocytes. While HFD has been studied in animal and clinical experiments, the majority of studies used non-traumatic bones to analyze its effects on bone. Hence, it is essential to study the effects of HFD on newly formed bone regeneration and consolidation following trauma.

Distraction osteogenesis (DO) which was used to make animal models in this study is a surgical technique that stimulates bone tissue regeneration by stretching tension forces on severed bone tissue (14). DO is a bone-regeneration process in which two vascularized bones are generated by gradual distraction, and as the bone segments are gradually distracted, new bone tissue is generated between them (14). Bone undergoes regeneration under controlled mechanical conditions owing to its intrinsic ability to do so (14). Additionally, DO is considered the best method for generating bone tissue *in vivo* and can be used to create a large new segment of bone tissue (15), which newly regenerated bone is more convenient to be observed and studied for the internal structural changes of bone regeneration than normal fracture. Hence, DO is widely used in experimental research of bone regeneration and clinical treatment that includes limb discrepancy, bone non-union, bone infection, bone defect, and malformation (16–20). Furthermore, in the process of bone regeneration, BMSCs differentiate into chondrocytes, fibroblasts, or osteoblasts to form fracture calluses, essential for healing fractured bones (21, 22). Hence, this study uses DO technique to establish an animal model that will enable us to observe bone regeneration more clearly following trauma.

According to our hypothesis, HFD has the potential to slow the rate and reduce the quality of bone tissue regeneration after trauma, and this result may be related to changes in the bone marrow microenvironment. To verify this hypothesis, a series of observations and tests were conducted in the fractured segments to assess the impact of HFD on bone regeneration and consolidation.

## 2 Materials and methods

### 2.1 Animals

**Figure 1:**
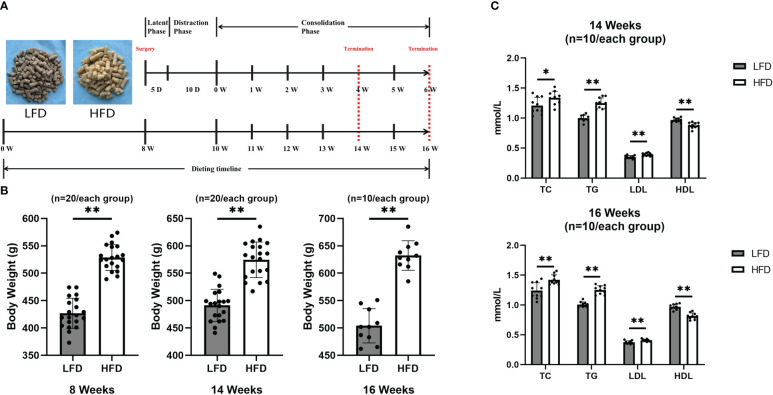
HFD-induced obese rats. **(A)**Dietary and surgical observation timeline. **(B)** Body weight values were higher in the HFD group at 8, 14, and 16 weeks than in the LFD group. **(C)** At 14 and 16 weeks of feeding, quantitative evaluations showed that TC, TG, and LDL levels in HFD were significantly higher than those in LFD while HDL levels were higher in LFD group. (*P<0.05, **P<0.01).

In this study, forty male Sprague Dawley (SD) rats (5 weeks old) were randomly divided into HFD group (n=20) and LFD group (n=20) as study subjects. The animals were raised at a temperature of 20-25°C and a humidity of 50-60% with free access to water and a pelleted diet of high-fat (HFD group, 60% kcal, D12492) or low-fat (LFD group, 4.5% kcal, General maintenance feed) (Beijing Boaigang Biotechnology Co. Ltd, Beijing, China) starting at 5 weeks of age. The time-dependent nodes of dietary and surgical observation have been shown in **Figure 1A**. All experimental procedures were approved by the Animal Ethics Committee of Xinjiang medical university (IACUC-202003318-82).

### 2.2 Surgical procedures and DO procedures

**Figure 2:**
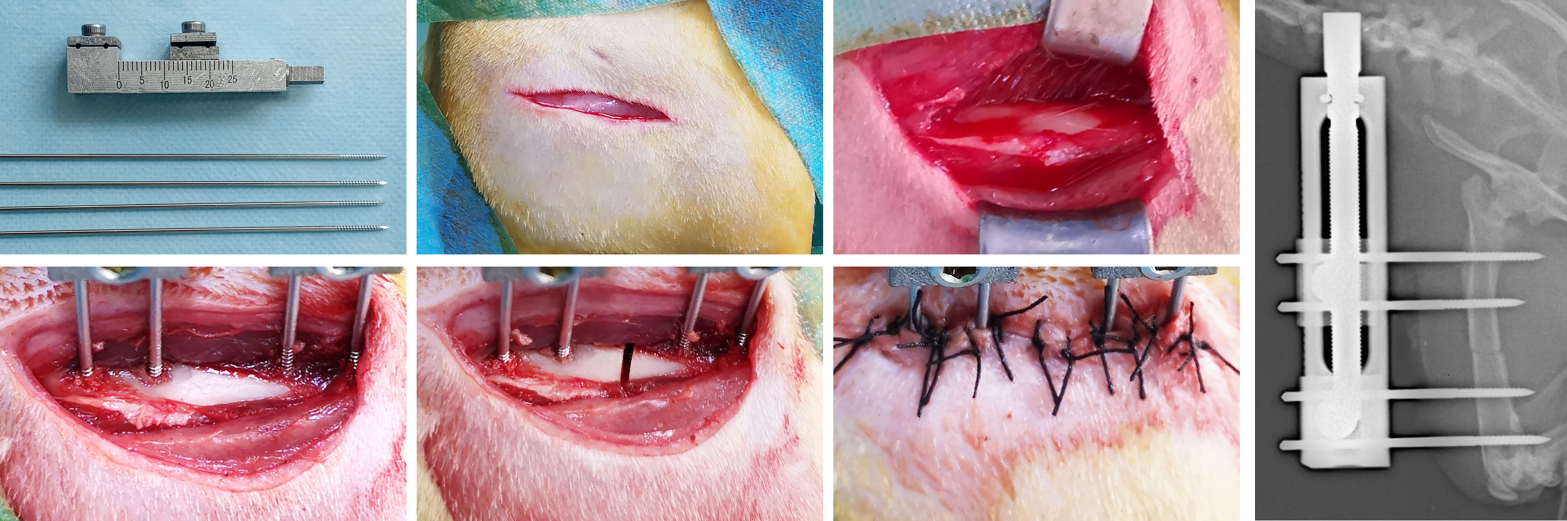
The surgical procedures for the rat right femur model of DO.

All surgical operations and postoperative procedures were performed by the same skilled surgical team. Eight weeks of feeding were administered to 40 rats before surgery. During the operation, anesthesia was administered using 2% pentobarbital sodium (3 mg/100 g) to each rat. For infection prevention, the preoperative administration of benzyl penicillin was carried out. Under sterile conditions, four stainless steel self-tapping screws were used to install a monolateral distraction external fixator (Designed and manufactured by this research team) on the right femur in rat, and then a mid-diaphysis transverse osteotomy was performed with the miniature bone saw (23; **Figure 2**).

With the antibiotic solution, daily pin site care was performed. Each experimental rat received daily intramuscular injections of benzyl penicillin for three days following surgery to prevent infection. Each rat was housed in a cage and allowed to move freely. Water and chow were provided (diets were the same as before surgery).

The DO procedure consisted of three phases (23, 24): a latency phase of 5 days, an active lengthening phase of 10 days (0.25 mm/12 h), and a consolidation phase of 42 days (six weeks).

Four and six weeks after consolidation, rats were randomly selected for sacrifice (n = 10 per group). Cardiac blood samples were drawn for lipid analysis. Bone samples were harvested from both femurs for further analysis.

### 2.3 Body weight and blood lipid analysis

Using a standard scale, the body weight was collected and analyzed in rats at the critical time points of 8, 14, and 16 weeks of feeding.Blood lipids including total cholesterol (TC), triglycerides (TG), low-density lipoprotein (LDL), and high-density lipoprotein (HDL) were determined by extracting heart blood from rats after 14 (four weeks of consolidation) and 16 (six weeks of consolidation) weeks of feeding.

### 2.4 Digital radiographic analysis

In order to monitor bone regeneration of the distraction zone, each rat was subjected to an anteroposterior (AP) radiographic examination weekly after isoflurane anesthesia until sacrifice using the same digital radiographic apparatus (HF400VA, MIKASA X-RAY Co., Ltd., Tokyo, Japan) and conditions (44kV, 4.5mAs). According to digital radiographic analysis, a callus formation stage is characterized by high fracture density, fuzzy fracture lines, and amorphous bone around the fracture. At this stage of healing, the callus disappears, fracture lines disappear, and trabeculae pass through the fractured end.

### 2.5 Micro-computed tomography (CT) analysis

Microstructural change within the distraction zone (bone regeneration) was quantitatively assessed using micro-CT imaging (80 kV, 313μA for 0.203 s, voxel size 18 μm; SkyScan 1176, Bruker, America) on the representative femur specimens (n=3 per group) that were collected at the 6-weeks of consolidation. Skyscan NRecon software was used to optimize and recompute the scanned images, and Skyscan CTAn software was used for three-dimensional (3D) analysis based on the manufacturer’s instructions. There was a definition of an area of interest (ROI) as the distraction zone surrounded by the periosteum at its proximal and distal ends (25). Bone mineral density (BMD) and bone volume/total tissue volume (BV/TV) measurements were made only on bone within the ROI.

### 2.6 Biomechanical test

In order to evaluate the strength of bone regeneration and repair, mechanical properties were used (n = 3 per group). In this procedure, samples of six-weeks consolidation without external fixators and screws were evaluated by a three-point bending test (RGM-3005T, ShenZhen Reger Instrument Co., Ltd., China), and control femurs consisted of unoperated femurs. In the experiments, with an 18mm span, the femur long axis was perpendicular to the blades. 0.5mm/min was constantly applied in the distraction zone with the AP direction until failure was achieved. Several indexes were measured on both the healthy and damaged femurs, including ultimate load, modulus of elasticity (E-modulus), energy to failure, and stiffness.

### 2.7 Histomorphometry in calcified tissue

For further analysis, a 10% formalin buffer was applied to all specimens for 48 hours, followed by an ethanol solution of 75%. Each group’s specimens (n = 3 per group) were successively dehydrated and fattened with xylene and embedded in methyl methacrylate following termination at each time point. With the help of a hard tissue microtome, sections 10μm thick were cut. In order to observe the histomorphometric appearance, Von Kossa, Masson Trichrome, Goldner Trichrome, and Safranin O staining were used.

### 2.8 H&E and immunohistochemistry in decalcified tissue

Four specimens per group were decalcified over a 4-week period using 10% ethylenediaminetetraacetic acid solution for evaluation of decalcified tissues. Following that, paraffin embedding was performed. For H&E and immunohistochemistry staining, five-mm sections were cut using a microtome. Observations were conducted on three ROI fields randomly selected for each section.

According to a standard protocol, the specimens were deparaffinized in xylene, rehydrated in gradient alcohol, and immunohistochemistry was administrated. For 20 minutes, the endogenous peroxidase activity was quenched with 0.3% hydrogen peroxide. An antigen retrieval solution of 0.4% pepsin was used for 25 minutes at 37°C, followed by a blocking solution containing 5% goat serum for 30 minutes at 37°C. Subsequently, anti-runt-related transcription factor 2 (RUNX2) (1:100, sc390351, Santa Cruz, CA, USA), anti-osterix (Osx) (1:400, ab209484, Abcam, Cambridge, UK), anti-osteocalcin (OCN) (1:100, 23418-1-AP, Proteintech, Wuhan, China), and anti-osteopontin (1:100, 22952-1-AP, Proteintech, Wuhan, China) primary antibodies were incubated overnight at 4°C on sections. The signals were detected with a horseradish peroxidase-streptavidin system (ZLI-9019, ZSGB-BIO, Beijing, China) after incubation in a secondary antibody (PV6000, ZSGB-BIO, Beijing, China) for 1h at 37°C, followed by hematoxylin counterstaining. For the analysis of each section, three fields of ROI were randomly selected and observed at a magnification of 200×. With Image Pro Plus 6.0 software, the same pixel value (brown) was set to calculate the positively stained areas in all specimens, and the proportion of positive area to total area was calculated by a semi-quantitative analysis.

### 2.9 Statistical analysis

OpenEpi V2 open source calculator was used to calculate sample size. A study with a two-sided 95% Confidence Interval and an 80% power required 20 animals per group. Statistical analysis was conducted using SPSS 22.0. A three-time calculation was performed under the same conditions for each data set to be analyzed. Throughout this paper, all continuous variables have been expressed as mean ± standard deviation (SD). An analysis of the Shapiro-Wilk test was conducted in order to determine the normality of the data. We evaluated the statistical differences between two specific groups by using the independent-samples t-test or the Mann-Whitney U test. P < 0.05 was considered a statistically significant difference. Based on GraphPad Prism v.6.0, graphs were created.

## 3 Results

### 3.1 Body weight and blood lipid analysis

Approximately eight weeks after feeding, there was a significant difference between groups in terms of body weight. Furthermore, the final results showed that the body weight was higher in the HFD group by 25% in 14 weeks and 30% in 16 weeks of feeding, compared with the LFD group (**Figure 1B**). Additionally, at the final observation, a blood lipid test was performed and showed that the HFD group was significant higher in TC, TG, and LDL than LFD group at 14 and 16 weeks of feeding (**Figure 1C**).

### 3.2 Sequential digital radiographs

In these experiments, there were no deaths among the rats, and they all recovered from the surgery and survived until the end of the experiment. There were no significant difficulties with daily activities for any of the rats, as they all achieved normal ambulation.

**Figure 3:**
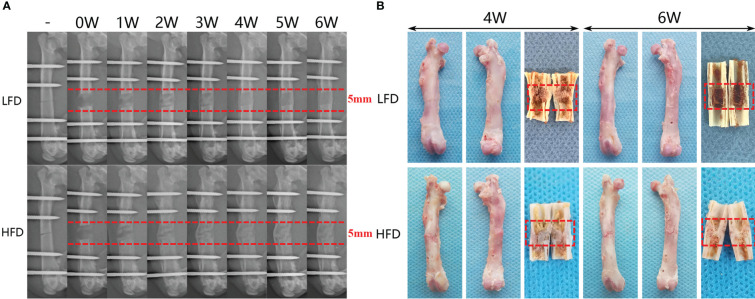
Distraction osteogenesis effects bone regeneration differently based on diet. **(A)** There is a six-week consolidation duration for the distraction X-ray images that regenerate each week. **(B)** After four- and six-week consolidation, a general image of the specimens can be seen.

**Figure 4:**
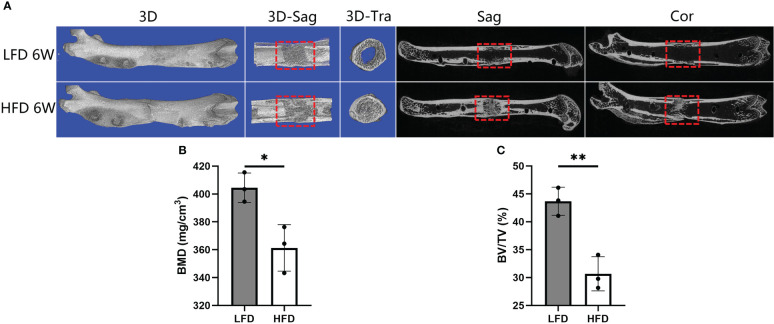
Results of micro-CT evaluation showing decreased regenerate quality after rats were fed a high-fat diet. **(A)** Representative three-dimensional (3D) micro-CT images of the distraction zone at the termination of the 6-week consolidation. **(B, C)** Quantitative evaluation of BMD and BV/TV, manifesting both two values in LFD group were significantly higher than those in HFD group. (*P<0.05, **P<0.01).

Digital radiographs were taken weekly to monitor the consolidation progress of distraction regeneration, as shown in **Figure 3**. During the first two weeks of the consolidation phase, high fracture density, fuzzy fracture lines, and amorphous bone around the fracture were presented in both two groups (**Figure 3A**). Nevertheless, bone regeneration in the LFD group was greater than in the HFD group after consolidation for three weeks. During the bone consolidation at week four and five, callus decrease and fracture lines disappear were observed in the LFD group but not in the HFD group. Additionally, in the distraction zone, there was a fuzzy fracture line in the HFD group after 6 weeks of consolidation, and proximal and distal fracture ends remained unhealed. However, the LFD group did not show this phenomenon, and the trabeculae pass through the fractured ends. Similarly, the general examination of dissected specimens and micro-CT examination after six weeks of consolidation revealed similar results (**Figures 3B**, **4**).

### 3.3 Three-dimensional (3D) microstructure of bone regeneration

Following 6 weeks of consolidation, the representative micro-CT images revealed almost complete recanalization of the marrow cavity in the LFD group, but a narrow closure of the bone marrow cavity was observed in HFD group (**Figure 4**). Additionally, a lower BMD was found in the HFD group (361.33 ± 16.71mg/cm^3^) compared to the LFD group (404.51 ± 10.54mg/cm^3^) (P=0.019) (**Figure 4B**). Similarly, there was a significant difference in BV/TV result between the two groups (30.67 ± 3.06% in HFD group vs. 43.67 ± 2.52% in LFD group) (P<0.01) (**Figure 4C**). According to the results, we hypothesized that in the HFD group, bone mineralization was significantly delayed and numerous non-mineralized tissues were presented in the distracted segments, which may contribute to a lower BMD and BV/TV in the HFD group compared with the LFD group.

### 3.4 Mechanical properties of regenerated bone

**Figure 5:**
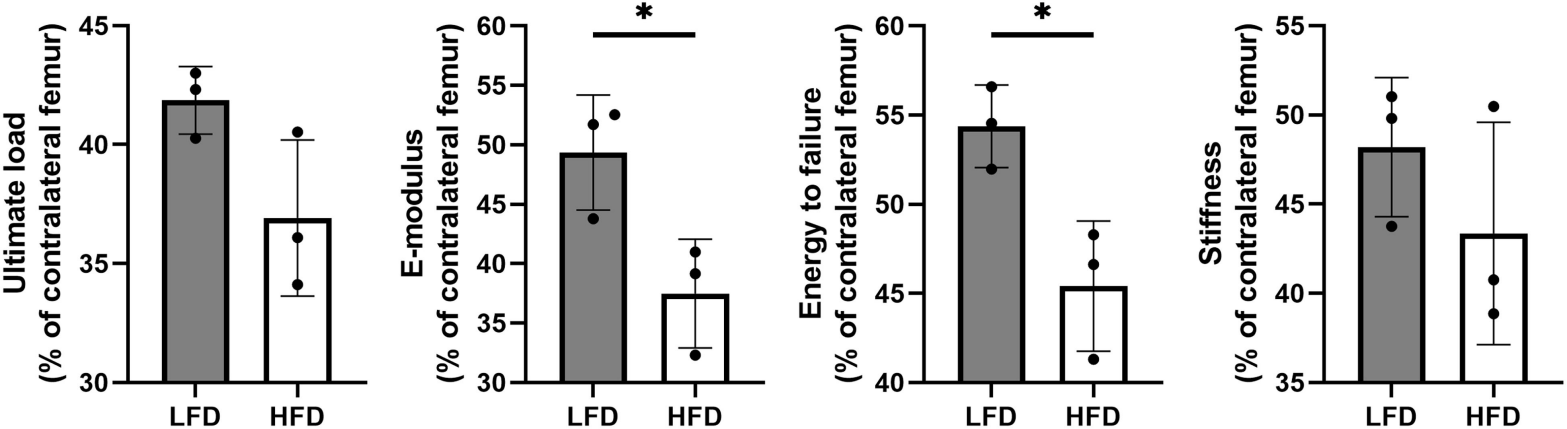
Results of mechanical properties and values were normalized to the contralateral femur. (*P<0.05).

Following 6 weeks of consolidation, the mechanical properties of collected samples were evaluated using a three-point bending test. The results showed that there were better outcomes in LFD group with a higher E-modulus (49.35 ± 4.84% in LFD group vs. 37.49 ± 4.57% in HFD group) and energy to failure (54.37 ± 2.32% in LFD group vs. 45.41 ± 3.65% in HFD group). However, no significant difference was observed in ultimate load (41.86 ± 1.42% in LFD group vs. 36.91 ± 3.28% in HFD group) and stiffness (48.19 ± 3.9% in LFD group vs. 43.36 ± 6.23% in HFD group) between the two groups (**Figure 5**).

### 3.5 Histomorphometry in calcified samples

**Figure 6:**
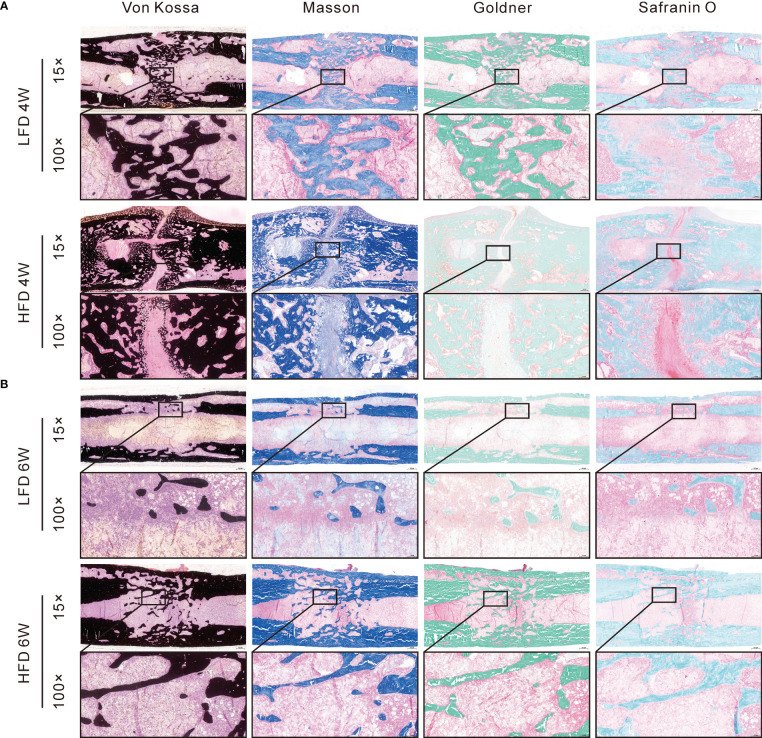
Histomorphological analysis of bone regeneration during the consolidation period. Von Kossa, Masson, Goldner Trichrome, and Safranin O & Fast Green staining indicated the decreased bone regeneration in HFD group. **(A)** 4-weeks consolidation. **(B)** 6-weeks consolidation.

Several histomorphological characteristics were examined in calcified samples, including Von Kossa, Masson, Goldner Trichrome, Safranin O & Fast Green. During Von Kossa staining, calcified tissue is black in color. In the HFD group, we can find an apparent gap in the interested area after 4 weeks of bony consolidation based on Von Kossa staining. Moreover, in the HFD group, Safranin O staining showed that there were numerous chondrocytes (stained red) in the interested area after 4 weeks of bony consolidation and indicated that there was still incomplete mineralization of regenerated bone here. However, in the LFD group, newly regenerated calluses were of higher quality and volume (**Figure 6**). At the 6 weeks of bony consolidation, Von Kossa staining showed that in both the LFD and HFD groups, the fracture space has disappeared, and the regenerated cortical bone is continuous. Complete reconstruction and recanalization of the bone marrow cavity were performed in the LFD group. However, in the HFD group, it has not yet been completely recanalized and there was still a small amount of callus in the marrow cavity. Similarly, according to Safranin O, Goldner Trichrome, and Masson staining, the observed results demonstrated that HFD significantly slows bone formation in DO (**Figure 6**).

### 3.6 Histological assessments in decalcified samples

**Figure 7:**
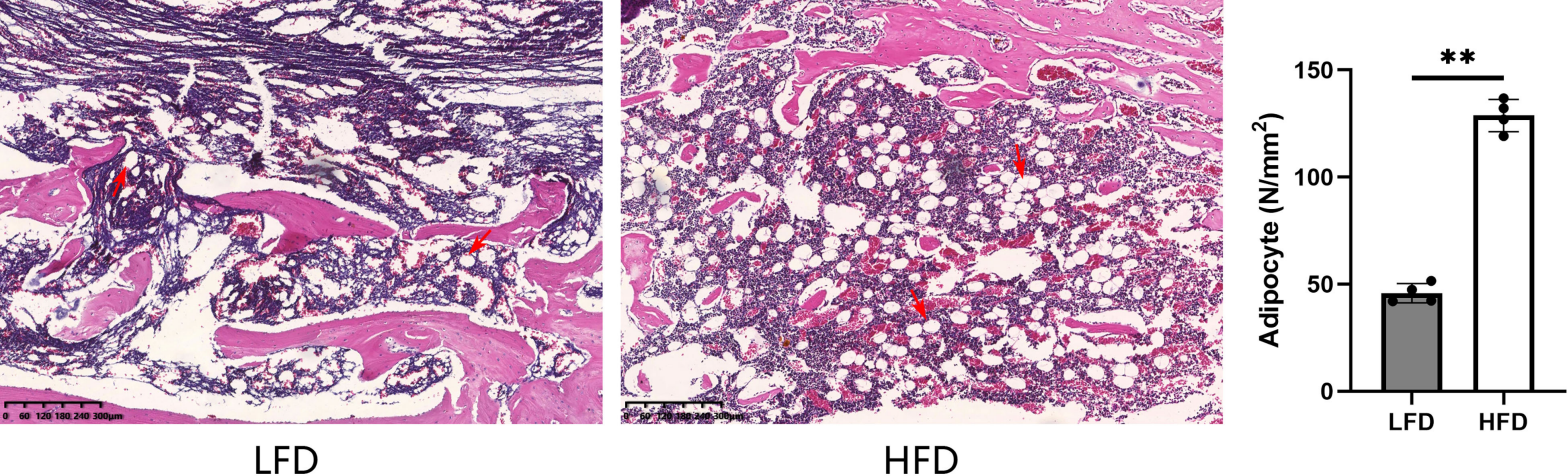
By H&E staining, the HFD group demonstrated significantly higher values than the LFD group when it came to bone marrow adipocytes. (**P<0.01, adipocytes were marked with red arrows).

At the end point of observation (6-weeks consolidation), H&E staining was used to assess the adipocytes in the bone marrow, and the qualitative results showed a significant difference between the HFD (128.8 ± 6.5 N/mm^2^) and LFD groups (45.8 ± 3.9 N/mm^2^). There is a higher level of adipose differentiation in the HFD group, according to the observation (**Figure 7**).

**Figure 8:**
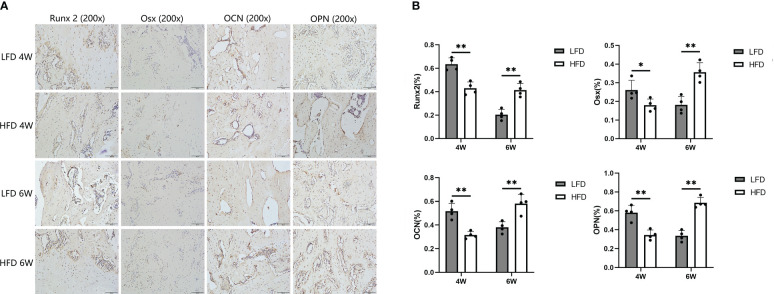
Immunohistochemical analysis. **(A)** Immunohistochemistry images of Runx 2, Osterix, OCN, and OPN in the two groups at the termination of 4-week and 6-week consolidation. **(B)** The semiquantitative measurements showed the 4 markers were highly expressed in LFD group compared to HFD group after 4 weeks of consolidation. At the termination of the 6-week consolidation, these indicators were lower expressed in LFD group compared to HFD group. (*P<0.05, **P<0.01).

In the immunohistochemical analysis, the expression of Runx2, Osterix, OCN, and OPN was lower in the HFD group at 4 weeks of consolidation compared with LFD group (P=0.039 or P<0.01). Interestingly, the aforementioned indicators were lower in the LFD group than in the HFD group at the end of the six-week consolidation period (P<0.01). According to our hypothesis, this may be because the regenerated trabeculae in the LFD group were more mature than those in the HFD group, and therefore, fewer osteogenic factors and proteins were produced in their place (**Figure 8**).

## 4 Discussion

The increased bone-forming activity that results from distraction is attributed to the stimulatory effect of tension on blood vessel formation and on the recruitment and proliferation of bone progenitor cells (26). In numerous previous studies, it has been shown that diet can affect the angiogenesis of regenerated bone tissue and the recruitment and proliferation of bone progenitor cells (6–8, 27). Additionally, there is evidence that HFD-induced high body mass and increased mechanical loading may benefit bone health (1–3). However, as a result of conflicting reports in previous studies on adipose tissue and bone regeneration, it has been difficult to assess the consequences of HFD on bone health (6–8, 27). In the present study, we compared the rate of regeneration of the femoral shaft in rats fed with different feeding methods and analyzed possible mechanisms behind it. As a result of HFD-rats, blood lipid levels rise, bone marrow adipose tissue (BMAT) is increased, and bone regeneration is reduced in the distraction gap.

According to previous research, HFD often results in weight gain, which undoubtedly benefits weight-bearing bones. As research has advanced, this view has gradually been refuted, and more and more researchers have confirmed that HFD is detrimental to bone development. Studies indicate that the effects of HFD on bone health increase with the duration of the diet. A study reported a significant increase in bone mass within 8 weeks of HFD in C57BL/6J mice, but a reduction in bone mass after 16 and 24 weeks of HFD (28). In addition, other studies showed that mice that were fed HFD for an extended period had a decreased bone mass, and that they had a poor recovery ability after LFD was performed (29, 30). The same results showed that HFD increased BMAT formation (as measured by increased volume, number, and size) and decreased bone mass (30–36). As Fazeli et al. confirmed, bone marrow adipocytes and osteoblasts had a negative correlation, so the differentiation direction of bone marrow mesenchymal stem cells (BMSCs) is crucial for bone regeneration. (37). In addition, other researchers demonstrated that bone marrow adipocytes secrete pro-inflammatory mediators (TNFα and IL-6) and adipokines, which can reduce the differentiative capacity of BMSCs in bone cells, but promote adipogenesis (38, 39).

Our study found that bone marrow adipocytes in the HFD group were significantly more numerous than those in the LFD group. However, it was observed that bone regeneration was delayed in the HFD group after 4 and 6 weeks of consolidation. The digital radiograph demonstrated that the bone regeneration after trauma was delayed in the HFD group. Furthermore, like radiographic results, the micro-CT examination revealed that LFD significantly improved bone regeneration and recanalization of the medullary cavity compared with HFD. Additionally, quantitative analyses concluded that bone quality is clearly impaired by HFD by decreasing BMD, BV/TV, and mechanical properties in regenerated segments. A slower bone regeneration was also observed in the HFD group when compared with the LFD group based on histomorphological assessment. In DO, HFD had detrimental effects on bone regeneration, as evidenced by the aforementioned compelling findings.

Bone health has been shown to be adversely affected by high-energy diets in previous studies (7, 30, 40–43). Similarly, our results showed that rats in the HFD group had less bone mass, slower bone regeneration, and higher BMAT volumes, suggesting that bone mass loss and slow bone regeneration are related to BMSCs differentiation and cytokines they secrete. Many researchers have reported an inverse relationship between bone formation and BMAT based on pathophysiological studies and suggested that enhanced differentiation of BMSCs into adipocytes reduces differentiation into osteoblasts, reducing bone formation as a result (32, 33, 44, 45). Our data support this conclusion under HFD, as we observed increased BMAT volume and quantity and delayed bone regeneration in the distraction zone in HFD rats.

Furthermore, hyperlipidemia load (TC, TG, and LDL) in the circulation increases lipid uptake by the skeleton and promotes the differentiation of BMSCs into adipocytes (46, 47). On the other hand, studies have shown that oxidation products of LDL-C can regulate the metabolic process of bone by affecting osteoblasts (48, 49). It has been shown that mildly modified LDL inhibits osteoblast differentiation by increasing extracellular oxidative stress responses among osteoblasts and BMSCs (50). Moreover, the oxidation products of LDL-C have also been found to stimulate the transformation of mouse BMSCs into adipocytes *in vitro* (51). Further, LDL-C and oxidized LDL can also reduce bone regeneration by stimulating p53 to cause osteoblast apoptosis (52). As a result of the above findings, the increased levels of LDL in the circulating blood appear to contribute to the delayed bone regeneration and mineralization in the distraction zone, which reduce the number and differentiation of osteoblasts and increase the number and activity of osteoclasts.

It is well known that bone tissue regeneration results from the joint regulation of bone formation and bone resorption. During bone formation, osteoblast differentiation plays a crucial role. Observation of osteogenic markers during bone regeneration can help determine osteoblast differentiation. In this study, these markers were analyzed and showed that a significant reduction in the expression of early and terminal osteogenic markers (Runx2 and Osx in early expression; OPN and OCN in a terminal expression) in the HFD group was observed. However, after 6 weeks of consolidation, the expression of these markers was reversed between the two groups, and there was a higher expression in the HFD group. According to the evaluation mentioned earlier, HFD significantly reduced bone formation. Hence, we speculated that in comparison to HFD group, less osteogenic relative factors and proteins were produced in the more mature regenerate trabeculae in LFD group at 6-week consolidation.

In bone formation, intramembranous and endochondral ossification play an important role, which requires a high blood supply (53). As bones develop and regenerate, blood supply and osteogenesis are tightly intertwined (54–57). As well as providing oxygen for bone development and regeneration, an adequate blood supply can also activate BMSCs and stimulate them to differentiate into osteoblasts (57). According to previous studies, increased BMAT impaired hematopoiesis, including depletion of B lymphocytes and inhibition of proliferation and differentiation of hematopoietic stem cells (58–60). Additionally, hyperlipidemia and the accumulation of lipid droplets in distraction zones may slow and obstruct local blood flow, impacting bone regeneration. (61). As a consequence of the above studies, the increase in BMAT observed in the distraction zone of rats receiving HFD may further affect bone regeneration by affecting the blood supply there.

Although our study yielded promising results, it also had several limitations. First of all, the present study only implemented one style of HFD, and future investigations are needed to identify whether additional styles will produce different results. In addition, different from humans, rats are walking with four limbs and receive less weight-bearing and mechanical stimulation on the femur. Therefore, further experimental methods need to be developed and optimized to avoid this difference. Moreover, in this study, an evaluation of the effectiveness of regeneration was based on histological and morphological characteristics of the regenerated bone, there may be future directions focusing on the molecular mechanisms underlying the effects observed. In summary, the observed results demonstrated that a relationship between HFD and bone regeneration may be mediated by the bone marrow microenvironment and dyslipidemia.

## 5 Conclusion

As a result of the establishment of a DO model in this study, the adverse effects of a high-fat diet on bone health were magnified and observed, and the mechanism may be related to the differentiation of BMSCs into adipose tissue under a high-fat diet. The result differs from traditional beliefs that high-fat diets are good for bone health because they cause weight gain. In addition, elevated lipid levels caused by a high-fat diet may affect the blood supply of new bone tissue, something that needs to be verified by further research.

## Data availability statement

The original contributions presented in the study are included in the article/**Supplementary Material**. Further inquiries can be directed to the corresponding authors.

## Ethics statement

The animal study was reviewed and approved by the Animal Ethics Committee of Xinjiang medical university.

## Author contributions

Conceptualization, FC, AY, YSL, and YL; methodology, FC and AY; software, FC; investigation, FC, AY, and KL; data analysis, FC, KL, WC, and RZ; writing original draft preparation, FC; review and editing, AY, KL, WC, RZ, YSL, and YL; supervision, YSL and YL; funding acquisition, AY, and YL. All authors contributed to the article and approved the submitted version.
